# Nonclinical Pharmacokinetics, Protein Binding, and Elimination of KBP-7072, an Aminomethylcycline Antibiotic, in Animal Models

**DOI:** 10.1128/AAC.00488-20

**Published:** 2020-05-21

**Authors:** Xiaojuan Tan, Min Zhang, Qingmei Liu, Ping Wang, Tian Zhou, Yuanju Zhu, Bin Chen, Meng Wang, Yong Xia, Vincent Benn, Fred Yang, Jay Zhang

**Affiliations:** a KBP Biosciences Co., Ltd., Jinan, China; b KBP Biosciences USA, Inc., Princeton, New Jersey, USA

**Keywords:** KBP-7072, pharmacokinetics, protein binding

## Abstract

KBP-7072 is a semisynthetic aminomethylcycline with broad-spectrum activity against Gram-positive and Gram-negative pathogens, including multidrug-resistant bacterial strains. The pharmacokinetics (PK) of KBP-7072 after oral and intravenous (i.v.) administrations of single and multiple doses were investigated in animal models, including during fed and fasted states, and the protein binding and excretion characteristics were also evaluated. In Sprague-Dawley (SD) rats, beagle dogs, and CD-1 mice, KBP-7072 demonstrated a linear PK profile after the administration of single oral and i.v. and multiple oral doses. The oral bioavailability ranged from 12% to 32%. The mean time to maximum concentration (*T*_max_) ranged from 0.5 to 4 h, and the mean half-life ranged from approximately 6 to 11 h. The administration of oral doses in the fed state resulted in marked reductions in the maximum plasma concentration (*C*_max_) and the area under the concentration-time curve (AUC) compared with dosing in fasted animals. The mean bound fractions of KBP-7072 were 77.5%, 69.8%, 64.5%, 69.3%, and 69.2% in mouse, rat, dog, monkey, and human plasma, respectively. Following a single 22.5-mg/kg oral dose of KBP-7072 in SD rats, the cumulative excretion in feces was 64% and that in urine was 2.5% of the administered dose. The PK results in animal models are consistent with single- and multiple-ascending-dose studies in healthy volunteers and confirm the suitability of KBP-7072 for once-daily oral and i.v. administration in clinical studies.

## Abstract (precis)

KBP-7072 is a semisynthetic aminomethylcycline with broad-spectrum activity against Gram-positive and Gram-negative pathogens, including multidrug-resistant bacterial strains. The pharmacokinetics (PK) of KBP-7072 after oral and intravenous (i.v.) administrations of single and multiple doses were investigated in animal models, including during fed and fasted states, and the protein binding and excretion characteristics were also evaluated. In Sprague-Dawley (SD) rats, beagle dogs, and CD-1 mice, KBP-7072 demonstrated a linear PK profile after the administration of single oral and i.

## TEXT

Bacterial infections represent a major burden of disease globally, with an impact on a substantial portion of the population (1). Increasing rates of resistance in recent years among Staphylococcus aureus, Streptococcus pneumoniae, Haemophilus influenzae, and many Gram-negative pathogens (2–4) pose serious challenges for providing effective treatment. Increased rates of resistance to macrolides (5) and beta-lactams (6) have been reported among S. pneumoniae isolates in the SENTRY program. Others have reported increased rates of bacterial resistance to beta-lactams, fluoroquinolones, macrolides, and older tetracyclines (7). The Centers for Disease Control and Prevention estimates that 2 million people in the United States develop an infection due to a resistant pathogen, accounting for >20,000 deaths per year (2). The rise in the incidence of infections caused by multidrug-resistant (MDR) bacteria poses a significant problem for patients with serious, potentially life-threatening infections because of the few available and effective treatment options (8, 9). The increased rate of resistance is associated with higher health care costs (10–12) and increased morbidity and mortality (10, 11). Bacterial resistance and potential side effects with some antibiotics, together with the substantial morbidity and mortality associated with resistant bacteria, highlight the need for new antibiotics to treat common infections.

KBP-7072 is a semisynthetic, aminomethylcycline antibiotic that inhibits the normal function of the bacterial ribosome. KBP-7072 exhibits a broad spectrum of *in vitro* antibacterial activity against Gram-positive and Gram-negative bacteria, including many multidrug-resistant pathogens. Notably, KBP-7072 is active against many of the bacteria causing respiratory infections, including S. pneumoniae, penicillin-resistant S. pneumoniae (PRSP), H. influenzae, Staphylococcus aureus, methicillin-resistant S. aureus (MRSA), and the atypical pathogens Mycoplasma pneumoniae, Legionella pneumophila, and Chlamydia pneumoniae, as well as MDR pathogens such as vancomycin-resistant enterococci (VRE), *Enterobacteriaceae* spp., Acinetobacter spp., and *Pseudomonas* spp. (13, 14). Results from single- and multiple-ascending-dose studies in healthy volunteers support the use of KBP-7072 administered once daily as an oral (p.o.) or intravenous (i.v.) formulation (15, 16).

We report the results from a series of studies that examined the pharmacokinetics (PK) of KBP-7072 in the CD-1 mouse, Sprague-Dawley (SD) rat, and beagle dog and during fed and fasted states as well as an evaluation of protein binding properties in animal and human plasma and excretion properties in the rat model.

## RESULTS

### Pharmacokinetics.

**FIG 1:**
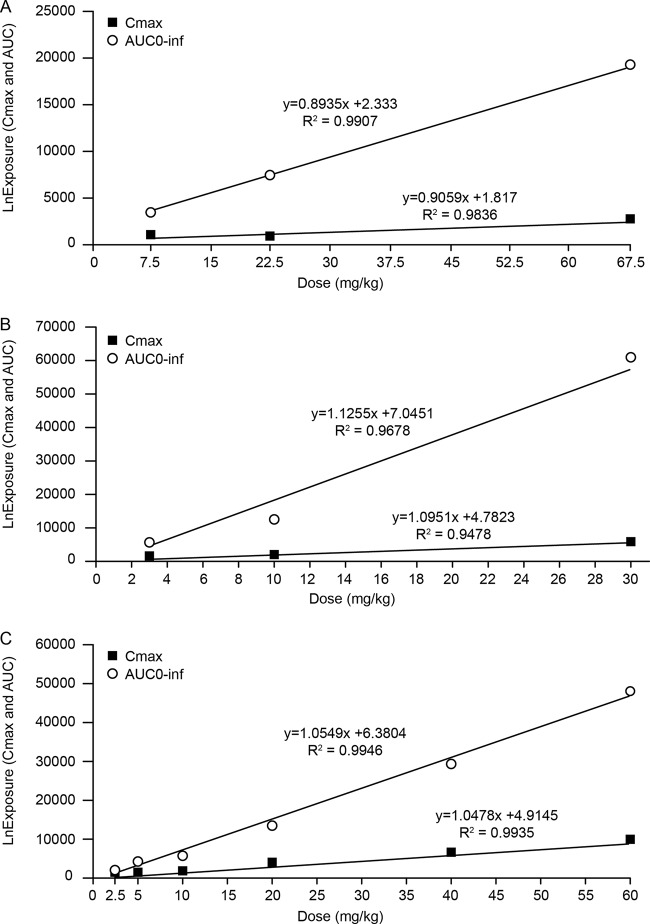
Linear regression of ln *C*_max_ (nanograms per milliliter) and ln AUC_0–∞_ (hours per nanograms per milliliter) versus dose (milligrams per kilogram) for KBP-7072 in SD rats after an oral dose of 7.5, 22.5, or 67.5 mg/kg (A); in beagle dogs administered a dose of 3, 10, or 30 mg/kg (B); and in CD-1 mice after a dose of 2.5, 5, 10, 20, 40, or 60 mg/kg (C).

**FIG 2:**
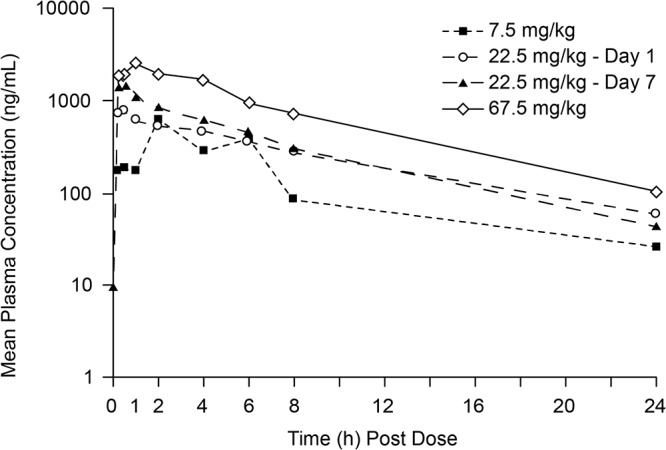
Mean plasma concentrations of KBP-7072 following a single oral dose of 7.5, 22.5, or 67.5 mg/kg or a once-daily dose of 22.5 mg/kg for 7 days in SD rats.

**(i) Sprague-Dawley rat.** KBP-7072 exhibited low clearance (CL) in rats. Following i.v. administration, the CL was 0.27 ± 0.037 liters/h/kg, the half-life was 10.8 ± 0.99 h, and the volume of distribution at steady state (*V*_ss_) was 3.0 ± 0.59 liters/kg (Table 1). Following oral administration, the bioavailability of KBP-7072 in rats ranged from 19.1% to 32.1% over a dose range of 7.5 to 67.5 mg/kg of body weight. After single oral administrations of KBP-7072, the mean time to maximum concentration (*T*_max_) ranged from 0.5 to 3.0 h, while the half-life ranged from 7.1 to 8.6 h. Across the dose range of 7.5 to 67.5 mg/kg, increases of both the maximum plasma concentration (*C*_max_) and the area under the concentration-time curve from time zero to infinity (AUC_0–∞_) were proportional to the dose (Fig. 1 and 2). *C*_max_ and AUC_0–∞_ values after multiple oral dosing were 1.8- and 1.1-fold higher than those after single oral dosing. The independent *t* test of PK parameters (*T*_max_, *C*_max_, AUC_0–_*_t_*, AUC_0–∞_, mean residence time from time zero to infinity [MRT_0–∞_], half-life, CL, and *V*_ss_) between male and female rats indicated no significant gender difference for PK parameters (*P > *0.05).

**TABLE 1:** Mean pharmacokinetic parameters for KBP-7072 following i.v. administration to SD rats and beagle dogs^*a*^

Parameter	Mean value ± SD (% RSD) for group	
	SD rats (*n* = 6)	Beagle dogs (*n* = 6)
Half-life (h)	10.8 ± 0.99 (9.2)	8.2 ± 0.90 (11.0)
Initial concn (ng/ml)	1,775 ± 418 (23.5)	4,250 ± 914 (21.5)
AUC_0–_*_t_* (h · ng/ml)	10,642 ± 1,544 (14.5)	28,786 ± 5,933 (20.6)
AUC_0–∞_ (h · ng/ml)	11,107 ± 1,512 (13.6)	29,286 ± 6,016 (20.5)
CL (liters/h/kg)	0.27 ± 0.037 (13.4)	0.11 ± 0.020 (18.5)
MRT_0–∞_ (h)	11.0 ± 0.72 (6.5)	11.3 ± 1.65 (14.6)
*V*_ss_ (liters/kg)	3.0 ± 0.59 (19.4)	1.2 ± 0.21 (18.1)

a The dose in rats was 7.5 mg/kg, and the dose in beagle dogs was 3 mg/kg. Values are means ± standard deviations and percent relative standard deviations (RSD). AUC_0–_*_t_*, area under the concentration-time curve from time zero to *t*; AUC_0–∞_, area under the concentration-time curve from time zero to infinity; CL, clearance; MRT_0–∞_, mean residence time from time zero to infinity; *V*_ss_, volume of distribution at steady state.

**FIG 3:**
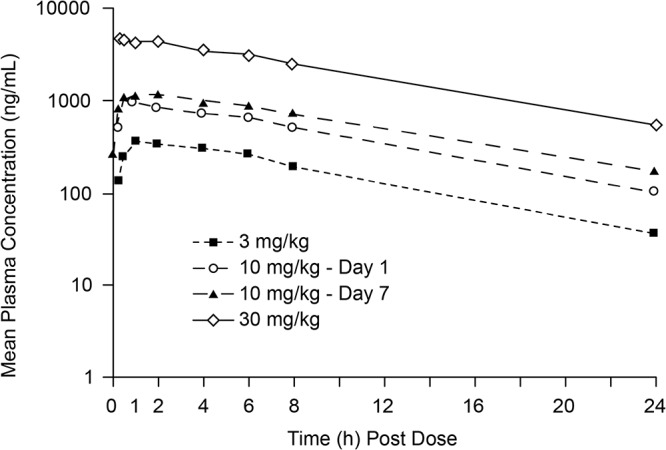
Mean plasma concentrations of KBP-7072 following a single oral dose of 3, 10, or 30 mg/kg or 10 mg/kg daily for 7 days in beagle dogs.

**(ii) Beagle dog.** Following i.v. administration, KBP-7072 exhibited low CL in dogs; the CL was 0.11 ± 0.020 liters/h/kg, the half-life was 8.2 ± 0.90 h, and the *V*_ss_ was 1.2 ± 0.21 liters/kg (Table 1). Following oral administration, the bioavailability of KBP-7072 in the beagle dog ranged from 11.9% to 20.8% over a dose range of 3 to 30 mg/kg (Table 2). After a single oral administration of KBP-7072, the mean *T*_max_ ranged from 1 to 3 h, while the half-life ranged from7.0 to 9.4 h. Across the dose range of 3 to 30 mg/kg, increases of both *C*_max_ and AUC_0–∞_ were proportional to the dose (Fig. 1 and 3). After oral administration for 7 days at 10 mg/kg, the plasma concentrations of KBP-7072 reached steady state on day 5, and no apparent drug accumulation was observed (Table 2). The independent *t* test of PK parameters (*T*_max_, *C*_max_, AUC_0–_*_t_*, AUC_0–∞_, MRT_0–∞_, half-life, CL, and *V*_ss_) between male and female dogs indicated no significant gender difference (*P > *0.05).

**TABLE 2:** Mean pharmacokinetic parameters for KBP-7072 following a single oral administration to SD rats and beagle dogs or multiple oral administrations (7 days) to beagle dogs^*a*^

Parameter	Mean value ± SD (% RSD) for group							
	SD rats given a single oral dose of:			SD rats given 22.5 mg/kg for 7 days (*n* = 8)	Beagle dogs given a single oral dose of:			Beagle dogs given 10 mg/kg for 7 days (*n* = 6)
	7.5 mg/kg (*n* = 6)	22.5 mg/kg (*n* = 6)	67.5 mg/kg (*n* = 6)		3 mg/kg (*n* = 6)	10 mg/kg (*n* = 6)	30 mg/kg (*n* = 6)	
Half-life (h)	8.4 ± 2.01 (24.0)	8.6 ± 2.66 (31.1)	7.1 ± 0.68 (9.6)	7.6 ± 1.24 (16.5)	8.3 ± 0.61 (7.3)	7.0 ± 1.31 (18.8)	9.4 ± 1.26 (13.5)	10.1 ± 1.67 (16.5)
*T*_max_ (h)	3	0.5	1	0.5	3	1	1	1
*C*_max_ (ng/ml)	719 ± 401 (55.8)	833 ± 316 (37.9)	2,707 ± 1,239 (45.8)	1,479 ± 238 (16.1)	468 ± 51 (11.0)	1,056 ± 260 (24.6)	5,918 ± 1,910 (32.3)	1,227 ± 365 (29.7)
AUC_0–_*_t_* (h · ng/ml)	3,481 ± 814 (23.4)	7,168 ± 1,822 (25.4)	18,910 ± 5,353 (28.3)	8,281 ± 1,481 (17.9)	4,412 ± 1,287 (29.2)	10,611 ± 2,083 (19.6)	59,136 ± 5,226 (8.8)	17,569 ± 3,527 (20.1)
AUC_0–∞_ (h · ng/ml)	3,563 ± 823 (23.1)	7,485 ± 1,787 (23.9)	19,047 ± 5,347 (28.1)	8,381 ± 1,474 (17.6)	4,515 ± 1,268 (28.1)	11,659 ± 2,313 (19.8)	60,837 ± 5,529 (9.1)	18,310 ± 3,888 (21.2)
MRT_0–∞_ (h)	8.0 ± 2.12 (26.5)	10.1 ± 2.81 (27.7)	7.8 ± 1.26 (16.2)	7.4 ± 0.84 (11.3)	10.0 ± 1.35 (13.6)	9.3 ± 1.70 (18.2)	11.4 ± 1.55 (13.6)	13.0 ± 2.67 (20.6)
*F* (%)	32.1 ± 7.41 (23.1)	22.5 ± 5.36 (23.9)	19.1 ± 5.35 (28.1)	25.2 ± 4.43 (17.6)	15.4 ± 4.3 (28.1)	11.9 ± 2.4 (19.8)	20.8 ± 1.9 (9.1)	18.8 ± 4.0 (21.2)

a Values are means ± standard deviations and percent relative standard deviations (RSD). AUC_0–_*_t_*, area under the concentration-time curve from time zero to *t*; AUC_0–∞_, area under the concentration-time curve from time zero to infinity; *F*, fraction (percent) absorbed; MRT_0–∞_, mean residence time from time zero to infinity; *T*_max_, time to maximum plasma concentration.

**(iii) CD-1 mouse.** After single-dose subcutaneous (s.c.) administration of KBP-7072 at doses of 2.5, 5, 10, 20, 40, and 60 mg/kg in CD-1 mice, the mean *T*_max_ ranged from 0.25 to 1 h, while the mean half-life ranged from 2.2 to 8.4 h (Table 3). Across the dose range of 2.5 to 60 mg/kg, *C*_max_ and AUC_0–∞_ were proportional to the dose (Fig. 1).

**TABLE 3:** Mean pharmacokinetic parameters for KBP-7072 after single s.c. doses in CD-1 mice (*n* = 3 per group)

Parameter	Value for KBP-7072 s.c. at a dose (mg/kg) of:					
	2.5	5	10	20	40	60
Half-life (h)	2.2	7.3	8.4	5.9	7.8	5.7
*T*_max_ (h)	0.5	0.25	1	1	1	0.25
*C*_max_ (ng/ml)	392	681	1,320	3,470	7,017	9,437
AUC_0–_*_t_* (h · ng/ml)	1,570	3,382	5,596	12,943	29,142	47,539
AUC_0–∞_ (h · ng/ml)	1,587	3,589	5,726	13,132	29,299	47,609

**(iv) Fed versus fasted state.** All animals were fasted prior to dosing on day 1 and day 7 and then fed between day 2 and day 6. Among SD rats given a single oral 22.5-mg/kg dose of KBP-7072 in the fasted and fed states, systemic exposure was markedly reduced in the fed state (Table 4). After oral administration of KBP-7072 at 22.5 mg/kg for 7 days, plasma concentrations of KBP-7072 at the same time points (predose and 1 h) on day 1 and day 7 were higher than those on days 4 to 6, indicating that administration with food reduced the absorption of KBP-7072 in the SD rat (Table 2). Among SD rats given oral KBP-7072 once daily for 7 days in either the fasted (20 mg/kg) or the fed (100 or 300 mg/kg) state, an independent *t* test of PK parameters between fasted and fed rat groups indicated that there was a significant difference (*P < *0.05). All PK parameters for KBP-7072 between fasted and fed SD rats (*T*_max_, *C*_max_, AUC_0–last_, and AUC_0–∞_) except for half-life showed lower exposure (AUC and *C*_max_) in fed rats (Table 5). These results indicate that the administration of an oral dose of KBP-7072 with food in SD rats reduces oral absorption, which significantly reduces systemic exposure (AUC and *C*_max_).

**TABLE 4:** Pharmacokinetic parameters in SD rats administered a single oral dose of KBP-7072 at 22.5 mg/kg in the fed and fasted states^*a*^

Parameter	Mean value for group ± SD (% RSD)	
	Fasted (*n* = 4)	Fed (*n* = 6)
Half-life (h)	6.8 ± 0.31 (4.5)	8.0 ± 1.55 (19.4)
*T*_max_ (h) (median)	0.38	3
*C*_max_ (ng/ml)	1,420 ± 161 (11.4)	86 ± 16 (18.8)
AUC_0–last_ (h · ng/ml)	9,220 ± 1,035 (11.2)	1,030 ± 245 (23.8)
AUC_0–∞_ (h · ng/ml)	9,292 ± 1,050 (11.3)	1,189 ± 267 (22.5)

a Values are means ± standard deviations and percent relative standard deviations (RSD). AUC_0–last_, area under the concentration-time curve from time zero to the last measurement; AUC_0–∞_, area under the concentration-time curve from time zero to infinity; *C*_max_, peak plasma concentration; *T*_max_, time to maximum plasma concentration.

**TABLE 5:** Pharmacokinetic parameters for KBP-7072 on day 1 and day 7 in the fed and fasted states in SD rats^*a*^

Parameter	Mean value ± SD for group					
	Fasted, 20 mg/kg (*n* = 3)		Fed, 100 mg/kg (*n* = 3)		Fed, 300 mg/kg (*n* = 3)	
	Day 1	Day 7	Day 1	Day 7	Day 1	Day 7
Half-life (h)	6.0 ± 0.38	6.0 ± 0.11	7.8 ± 1.37	7.4 ± 0.54	9.6 ± 2.01	7.6 ± 0.34
*T*_max_ (h) (median)	0.5	0.5	4	1	2	1
*C*_max_ (ng/ml)	1,037 ± 21	507 ± 78	306 ± 203	1,435 ± 753	1,174 ± 371	3,675 ± 1,357
AUC_0–last_ (h · ng/ml)	7,378 ± 782	4,100 ± 1,011	4,288 ± 2,807	10,743 ± 5,194	14,253 ± 2,623	35,557 ± 11,169
AUC_0–∞_ (h · ng/ml)	7,790 ± 798	4,342 ± 1,048	4,761 ± 2,866	11,815 ± 5,588	17,263 ± 3,446	39,720 ± 12,292
AUC_0–last_/dose	369	205	42.9	107	47.5	119

a Values are means ± standard deviations. AUC_0–last_, area under the concentration-time curve from time zero to the last measurement; AUC_0–∞_, area under the concentration-time curve from time zero to infinity; *C*_max_, peak plasma concentration; *T*_max_, time to maximum plasma concentration.

After a 7-day regimen of oral KBP-7072 at 100 and 300 mg/kg in fed rats and at 20 mg/kg in fasted rats, the exposure versus dose in fasted rats was considerably higher than that in fed rats. Although the AUC_0–last_/dose ratio was 369 after the first dose in fasted rats at 20 mg/kg and was higher than the AUC_0–last_/dose ratio (107 at 100 mg/kg and 119 at 300 mg/kg) after the day 7 dose in fed rats, the exposure of fasted rats was decreased, while the exposure of fed rats was increased as the dosing days increased.

### Protein binding.

Plasma stability tests of KBP-7072 at concentrations of 0.2, 2, and 20 μM at 37°C showed that KBP-7072 was stable for 4 h in mouse, rat, dog, monkey, and human plasma, with ≥90.4% of KBP-7072 remaining after the 4-h incubation. The mean bound fractions of KBP-7072 were 77.5%, 69.8%, 64.5%, 69.3%, and 69.2% in ICR mouse, SD rat, beagle dog, cynomolgus monkey, and human plasma, respectively. The bound fraction remained stable across drug concentrations of 0.2, 2, and 20 μM.

### Excretion.

Following a single 22.5-mg/kg oral dose of KBP-7072 in SD rats, the cumulative excretion over 96 h in feces was 64% and that in urine was 2.5% of the administered dose. Most excretion occurred within 48 h after the dose.

## DISCUSSION

Results from the studies of the PK of KBP-7072 in animal models showed a *T*_max_ of 0.5 to 4 h and a half-life of approximately 6 to 11 h after oral or i.v. administration. Exposures (*C*_max_ and AUC) were dose proportional across a range of oral doses, with low intersubject variability, and no significant differences were observed for the PK of KBP-7072 between male and female animals. Less than 2-fold accumulation occurred for AUC and *C*_max_ when KBP-7072 was administered as a single oral 10-mg/kg dose or as multiple daily doses for 7 days in beagle dogs. Similar findings were observed in healthy volunteers given single oral doses of KBP-7072 at 30 to 300 mg and multiple daily doses of 100 or 200 mg for 10 days (15, 16). KBP-7072 exposure (*C*_max_ and AUC) increased in an approximately dose-proportional manner, with a *T*_max_ of 1.5 to 2 h, although in healthy volunteers, the half-life was >24 h (15, 16). Some accumulation occurred with multiple doses (16). A dose-proportional increase in exposure was observed with KBP-7072 in a murine model of pneumonia (17). In this study, the mean 24-h doses to achieve bacteriostatic and bactericidal activity (24-h *f*AUC [AUC for the free, unbound fraction of the drug]/MIC) against S. pneumoniae were 0.09 and 0.39 mg/kg, respectively (17).

In separate studies in SD rats, an oral dose of KBP-7072 in fed animals resulted in marked decreases in *C*_max_ and AUC compared with animals that were fasted overnight. In healthy volunteers, oral administration of KBP-7072 with food also delayed absorption and reduced exposure (*C*_max_ and AUC) compared with dosing in fasted subjects (15, 16). This is consistent with results from oral administration of omadacycline, where dosing with food markedly reduced systemic exposure (18). It is important to investigate the effect of antibiotics on the gut microbiota. Studies of KBP-7072 have not been done yet; however, its effects on the gut microbiota will be evaluated in future studies.

Protein binding for KBP-7072 was approximately 75% in animal and human plasma. This compares with >80% for eravacycline (19), approximately 20% for omadacycline (16, 18), and 71% to 89% for tigecycline when assessed in animal plasma (20). The bioavailability of KBP-7072 ranged from 19% to 28%, compared with 22.6% in a rat model with omadacycline (21). Urine recovery of KBP-7072 was 2.5%, compared with 14% after oral and 27% after i.v. administration of omadacycline and 34% and 33% after i.v. administration of eravacycline and tigecycline, respectively (19–21).

KBP-7072 is undergoing clinical development for the treatment of community-acquired bacterial pneumonia (CABP) and other serious infections due to Gram-positive and Gram-negative aerobes, including many multidrug-resistant pathogens. *In vitro*, KBP-7072 demonstrated MIC_90_ values of <1 μg/ml across a range of pathogens, including typical and atypical pathogens associated with CABP (13, 14, 22). In a murine mouse model of pneumonia, peak KBP-7072 concentrations ranged from 0.12 to 25.2 μg/ml (17). Epithelial lining fluid concentrations of KBP-7072 were 82% to 238% of plasma concentrations. At 24 h, plasma AUC/MIC values for a 2-log_10_ kill were 7.2 and 31.4 for Staphylococcus aureus and Streptococcus pneumoniae. Thus, the doses used in this study were severalfold higher than the ones previously reported to produce static and cidal effects (17). Results from these studies of the PK of KBP-7072 in animals support once-daily administration of KBP-7072 and are consistent with findings from single- and multiple-dose studies of KBP-7072 in healthy volunteers where the elimination half-life exceeded 24 h (15, 16). These results together with results from *in vitro* studies of microbiological activity suggest that KBP-7072 is a promising antibiotic with the potential to expand the armamentarium of drugs available to treat serious infections, especially in an era of growing bacterial resistance to antimicrobials.

## MATERIALS AND METHODS

All *in vivo* studies were conducted under appropriate Institutional Animal Care and Use Committee-approved protocols and in accordance with KBP Biosciences Institutional Animal Care and Use Committee guidelines. Male and female adult SD rats weighing 200 to 300 g and female 6- to 8-week-old CD-1 mice weighing 25 ± 2 g were obtained from Vital River Laboratories, Beijing Co., Ltd., China. Male and female beagle dogs at least 6 months old and weighing 8 to 10 kg were obtained from Beijing Marshall Biotechnology Co., Ltd., China. Animals were housed in a segregated pathogen-free room under controlled temperature, humidity, airflow, and lighting conditions and were fed standard food and water.

KBP-7072 was freshly prepared prior to each study, and final concentrations were determined prior to dosing. Following dosing, blood samples were obtained at frequent intervals through 48 h to measure plasma KBP-7072 concentrations. Concentrations of KBP-7072 in plasma were determined using a validated liquid chromatography-tandem mass spectrometry (LC-MS/MS) method (data on file at KBP Biosciences Co., Ltd., Jinan, China).

### Pharmacokinetics. (i) SD rat.

Pharmacokinetic studies of KBP-7072 were conducted in male (*n* = 17) and female (*n* = 17) SD rats. Animals received a single 7.5-mg/kg i.v. dose; single oral doses of 7.5, 22.5, and 67.5 mg/kg; or multiple oral doses of 22.5 mg/kg once daily for 7 days. Animals had free access to water but were fasted for 12 h prior to dosing. In the multiple-dose group, animals were fasted for 12 h prior to dosing on days 1 and 7, and food was withheld until 4 h after dosing.

### (ii) Beagle dog.

Pharmacokinetic studies of KBP-7072 were conducted in male (*n* = 3) and female (*n* = 3) beagle dogs. Animals received KBP-7072 as a single 3-mg/kg i.v. dose; single oral doses of 3, 10, and 30 mg/kg; and multiple oral doses of 10 mg/kg once daily for 7 days. Animals were fasted for 12 h prior to dosing, and food was supplied 4 h after dosing.

### (iii) CD-1 mouse.

This study characterized the PK of KBP-7072 in female CD-1 mice (*n* = 18). Animals received KBP-7072 as a single s.c. dose of 2.5, 5, 10, 20, 40, or 60 mg/kg. Animals were provided food and fresh drinking water throughout the study.

### Food effect.

In one study, SD rats (6 males and 6 females) either were fasted over 12 h prior to dosing, and food was withheld until 4 h after KBP-7072 administration, or were fed as usual. A single 22.5-mg/kg oral dose of KBP-7072 was administered in the fasted or fed state to each animal. In a second study, SD rats either were fasted over 12 h prior to dosing, and food was withheld until 4 h after KBP-7072 administration, or were fed as usual. Animals received oral KBP-7072 at 20 mg/kg once daily for 7 days (fasted, *n* = 3), oral KBP-7072 at 100 mg/kg once daily for 7 days (fed, *n* = 3), or KBP-7072 at 300 mg/kg once daily for 7 days (fed, *n* = 3). Blood samples were collected on day 1 and day 7 at 15 and 30 min and 1, 2, 4, 8, and 24 h after the KBP-7072 dose.

### Protein binding.

Binding of KBP-7072 to mouse, rat, dog, monkey, and human plasma proteins was determined *in vitro* using equilibrium dialysis. Fresh blood samples were obtained from animals and centrifuged to separate plasma, and plasma samples were stored at −80°C until used. KBP-7072 was prepared from a stock solution of 10 mM in dimethyl sulfoxide. The time to dialysis equilibration used was 4 h, where KBP-7072 was found to be stable in plasma after a 4-h incubation at 37°C. Plasma protein binding studies were performed using a 96-well HTDialysis equilibrium dialysis chamber apparatus. An aliquot (100 μl) of KBP-7072-spiked plasma was added to the donor side of each designated well. An equal volume of phosphate buffer (containing 0.002% Tween 80) was added to the receiver side. The plate was covered with adhesive sealing film to prevent evaporation and placed in a water bath at 37°C for 4 h with a shaking speed of 80 rpm. The time course of protein binding equilibration was assessed in triplicate in human plasma after 2, 4, 6, and 8 h of incubation at 37°C, with a KBP-7072 concentration of 2 μM. Based on the time course of binding equilibrium, the subsequent cross-species assessment of the bound fraction was conducted with 4-h equilibration in mouse, rat, dog, monkey, and human plasma at KBP-7072 concentrations of 0.2, 2, and 20 μM.

### Excretion.

SD rats (3 males and 3 females) received a single oral 22.5-mg/kg dose of KBP-7072 after an overnight 12-h fast, and food was supplied 4 h after dosing. Urine and feces were collected predose and at intervals of 0 to 24, 24 to 48, 48 to 72, and 72 to 96 h. Concentrations of KBP-7072 were determined with a validated LC-MS/MS method.

### Analytical method.

Analysis of all plasma KBP-7072 samples was performed with a validated LC-MS/MS method on a Shimadzu LC-20AD pump interfaced with an AB Sciex (MA, USA) API4000 mass spectrometer using an Agilent Zorbax SB C_18_ (50 by 2.1 mm, 5.0 μm) or an XBridge C_18_ (4.6 by 150 mm, 5 μm) column. For animal PK plasma sample analysis, 20-μl animal plasma samples were mixed with 20 μl of phosphate-buffered saline (PBS) (100 mM, pH 7.4) and 160 μl of a 0.1% formic acid-methanol solution with an internal standard (IS). One hundred microliters of the supernatant was added to 96-well plates, which contained 100 μl water after being centrifuged at 12,000 rpm for 5 min. Twenty microliters of this solution was injected into the LC-MS/MS system for analysis.

For protein binding sample analysis, at each time point, aliquots were taken from each side of each well and diluted appropriately with either buffer or blank plasma. Fifty microliters from the plasma side was diluted with 50 μl of phosphate buffer (containing 0.002% Tween 80). Fifty microliters of the buffer side was diluted with 50 μl of blank plasma. All the samples were added to a 10-μl EDTA-K_2_ solution. Samples for analysis were prepared by the addition of 500 μl of acetonitrile containing 50 ng·ml^−1^ of the IS. After vortexing, the samples were centrifuged at 13,000 rpm for 8 min. Seventy microliters of the supernatant in each tube was transferred and diluted with an equal volume of water. An aliquot (3 μl) of each sample was injected into the LC-MS/MS system for analysis.

For analysis of urine and fecal samples, 100 μl of urine or fecal homogenate samples was mixed with 200 μl of PBS (100 mM, pH 7.4) and 600 μl of a 1% formic acid-methanol solution containing the internal standard. One hundred microliters of the separated supernatant was transferred into 96-well plates, which contained 100 μl of water after being centrifuged for 5 min at 15,294 relative centrifugal force (rcf). A 10-μl aliquot was injected into the LC-MS/MS system for analysis.

### Statistical analysis.

Data were acquired using Analyst v1.6.1 software (AB Sciex). The means, standard deviations, coefficients of variation, and other parameters were calculated by using Microsoft Office Excel 2007. All pharmacokinetic parameters were calculated using the noncompartmental model of Pharsight Phoenix 6.3. Percent bioavailability (*F*%) was calculated using the formula *F*% = [AUC_0–∞_ (p.o.) × dose (i.v.)]/[AUC_0–∞_ (i.v.) × dose (p.o.)] × 100.
